# Genetic Diversity of *Rhipicephalus (Boophilus) microplus* for a Global Scenario: A Comprehensive Review

**DOI:** 10.3390/pathogens13060516

**Published:** 2024-06-18

**Authors:** Muthu Sankar, Binod Kumar, Haranahally Vasanthachar Manjunathachar, Balasamudram Chandrasekhar Parthasarathi, Abhijit Nandi, Chemmangat Kunnath Subramanian Neethu, Gaurav Nagar, Srikant Ghosh

**Affiliations:** 1 Entomology Laboratory, Division of Parasitology, Indian Council of Agricultural Research-Indian Veterinary Research Institute, Izatnagar, Bareilly 243122, India; drsankarm@gmail.com (M.S.); csneethu50@gmail.com (C.K.S.N.); gauravn19@gmail.com (G.N.); 2 Department of Veterinary Parasitology, College of Veterinary and Animal Sciences, Bihar Animal Sciences University, Kishanganj 855107, India; drkumarbinod@gmail.com; 3 Indian Council of Medical Research-National Institute of Traditional Medicine, Department of Health Research, Govt. of India, Nehru Nagar, Belagavi 590010, India; manjunathachar632@gmail.com; 4 Livestock Research Station, Sri Venkateswara Veterinary University, Palamaner 517408, India; parthb763@gmail.com; 5 Department of Veterinary Parasitology, West Bengal University of Animal and Fishery Sciences, Kolkata 700037, India; drabhijitnandi@gmail.com; 6 Indian Veterinary Research Institute, Eastern Regional Station, 37, Belgachia Road, Kolkata 700037, India

**Keywords:** genetic diversity, cytochrome c oxidase I, ribosomal genes, microsatellites, Bm86, sodium channel genes, *Rhipicephalus microplus*

## Abstract

*Rhipicephalus microplus* poses a substantial threat to livestock health and agricultural economies worldwide. Its remarkable adaptability to diverse environments and hosts is a testament to its extensive genetic diversity. This review delves into the genetic diversity of *R. microplus*, employing three pivotal genetic markers: the cytochrome c oxidase I (COX1) gene, ribosomal genes, and microsatellites. The COX1 gene, a crucial tool for genetic characterization and phylogenetic clustering, provides insights into the adaptability of ticks. Ribosomal genes, such as internal transcribed spacer regions (ITS-1 and2) as well as 18S and 28S, are routinely utilized for species differentiation. However, their use is limited due to indels (insertions and deletions). Microsatellites and minisatellites, known for their high polymorphism, have been successfully employed to study populations and genetic diversity across various tick species. Despite their effectiveness, challenges such as null alleles and marker variations warrant careful consideration. Bm86, a well-studied vaccine candidate, exhibits substantial genetic diversity. This diversity directly influences vaccine efficacy, posing challenges for developing a universally effective Bm86-based vaccine. Moreover, the review emphasizes the prevalence of genes associated with synthetic pyrethroid resistance. Identifying single nucleotide polymorphisms in the acaricide-resistant genes of *R. microplus* has facilitated the development of molecular markers for detecting and monitoring resistance against synthetic pyrethroids. However, mutations in sodium channels, the target site for synthetic pyrethroid, correlate well with the resistance status of *R. microplus*, which is not the case with other acaricide target genes. This study underscores the importance of understanding genetic diversity in developing effective tick management strategies. The choice of genetic marker should be tailored based on the level of taxonomic resolution and the group of ticks under investigation. A holistic approach combining multiple markers and integrating additional molecular and morphological data may offer a more comprehensive understanding of tick diversity and relationships. This research has far-reaching implications in formulating breeding programs and the development of vaccine against ticks and tick-borne diseases (TTBDs) as well as strategies for the management of resistant ticks.

## 1. Introduction

Genetic diversity is a nucleotide or nucleotides’ change in a gene or group of genes within a species population. It is a natural process for improving environmental adaptability [1]. Genetic diversity influences not only environmental adaptation but also adaptation to a wide range of hosts. *Rhipicephalus microplus*, known as the southern cattle tick, is one of the most invasive and adapted ectoparasites reported in many regions of the world [2,3,4]. It tends to replace other tick species and subgenera of the same species [2,5,6,7]. This phenomenon is attributed to improved animal husbandry practices which are unsuitable for the survival of two or three host ticks [8]. In addition, animal trading is also a factor in accelerating the spread of *R. microplus* in various countries [9]. Most importantly, adapting *R. microplus* in different geographical areas is associated with the speedy development and spread of acaricide resistance [2,10]. *R. microplus* infestations directly cause substantial economic losses to cattle owners compared with infestations of *R. decoloratus* and *R. annulatus* by sucking the blood of cattle, causing skin damage, and acting as a vector for deadly pathogens, causing babesiosis and anaplsomosis [11,12,13].

Traditionally, tick species have been identified based on morphological parameters [14,15,16]. However, identification of the *R. microplus* species complex is more complex, and it is challenging to differentiate the taxa, namely *R. microplus* clade A *sensu*, *R. microplus* clade B *sensu* (Burger et al. (2014)) [17], *R. microplus* clade C *sensu* (Low et al. (2015)) [18], *R. annulatus*, and *R. australis*. *R. annulatus* is found in southern Europe, south, western, and central Asia, northern and tropical sub-Saharan Africa, Mexico, and the border regions of Texas in the USA [19]. *R. australis* has been reinstated to describe *R. microplus*, which is found in Australia, New Caledonia, and certain parts of Southeast Asia [20,21]. Clades A and B represent *R. microplus* ticks from Asia, South America, and Africa, while clade C is primarily distributed in the Indian subcontinent, southern China, and Malaysia [17,18]. Thus, molecular markers are warranted to differentiate closely related species and subspecies taxonomically. This information may provide insight into the population structure and genetic variability of the *R. microplus* complex and its ability to transmit diseases [17,22]. Observable differences in traits among species arise from genetic variations in coding or non-coding regions, collectively known as polymorphisms [23]. Molecular genetic markers, which are located at specific loci within the genome, are where these DNA variations occur. They play a crucial role in distinguishing closely related species at both intra- and interspecific levels [24,25].

*R. microplus* is reported to have originated in India and disseminated to other parts of the world through animal trading. Studies on the genetic diversity in *R. microplus* strains across countries have been conducted by various researchers using mitochondrial and ribosomal genes (16S rRNA, 12S rRNA, COX1, microsatellites, and internal transcribed spacer) [17,18,22,26,27,28,29,30,31,32,33,34,35]. The results of these studies exhibited different clades and the existence of crypto species of *R. microplus* in different parts of the globe [17,18,34]. However, the presence of indels (insertions and deletions) in ribosomal (12S and 16S) genes may be a limiting factor during sequence alignments [36]. Other genes like ITS-2 and the 5.8S and 28S ribosomal subunits of rDNA have also been used for genetic diversity analysis in parasitology [37]. However, the ITS-2-based marker did not show any conclusive results among the *R. microplus* strains [21].

Moreover, genetic diversity in protein coding genes, Bm86, a vaccine candidate against *R. microplus* and synthetic pyrethroid resistance-conferring genes, and sodium channels are also included here. Bm86-based commercial vaccines are available underthe trade names of TickGARD^®^ (Hoechst, Australia), TickGARD PLUS^®^ (Intervet, Australia), and Gavac^®^ (Heber Biotec, Havana, Cuba) [37,38]. However, the efficacy of commercial vaccines varies across geographical areas [39,40,41,42] due to variations and polymorphism in the Bm86 gene, and this is one of the major impediments to its commercializing globally as an anti-*R. microplus* vaccine. Genetic variations in Bm86 have resulted in many haplotypes in various geographical regions [39,40,41,42], and these forced researchers to make vaccines using region-specific strains.

Extensive application (and arguably misuse) of acaricides led to the development of resistance populations [43]. Resistance is an inherited trait or acquired resistance probably expressed due to the selection pressure upon acaricide treatment. Single-nucleotide polymorphisms on target genes are one of the mechanisms responsible for developing acquired resistance to chemical acaricides [43]. In the sodium channel gene, multiple mutations have been reported in different parts of the world. Therefore, understanding genetic diversity and its relationship with prevalence is pivotal to implementing control programs. This review discusses the suitability of various markers for genetic diversity studies amongst *R. microplus* strains and their importance in the management of TTBDs.

## 2. Diversity in the Marker Genes

### 2.1. Cytochrome c Oxidase I (COX1)

**Figure 1:**
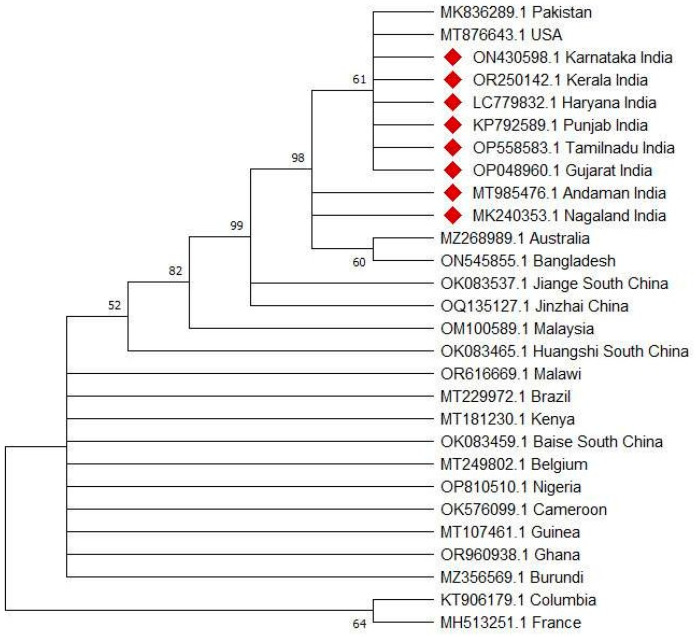
Phylogentic analysis based on COX1 gene sequences of *Rhipicephalus microplus*.

This gene is widely accepted for genetic characterizations and phylogenetic clustering of *R. microplus* [28]. COX1 has been used as a DNA marker in DNA barcoding [44] to portray and identify species. The superiority of the COX1 gene has already been shown in differentiating the *R. microplus* complex compared with other genes, like 12S, 16S, or the ITS2 region [17,18,23]. Phylogenetic analysis of the COX1gene of *R. microplus* from various countries revealed the prevalence of five distinct clusters, which include *R. microplus* clade A *sensu* (belonging to Africa, Asia, and South America), *R. microplus* clade B *sensu* (belonging to southern China and northern India) [17], *R. microplus* clade C *sensu* (belonging to Malaysia and India) [18], *R. australis*, and *R. annulatus* [17,18]. Phylogenetic analysis ofCOX1 using representative sequences from various countries revealed a similar pattern (Figure 1). For phylogenetic reconstruction, the sequences of COX1genes from different parts of the world were retrieved from the GenBank database and analyzed using MEGA-11. The best fit nucleotide substitution model was predicted using MEGA-11. A phylogenetic tree was constructed using the maximum likelihood method and a Tamura–Nei model using 1000 bootstrap replications to confirm the authenticity of the taxa analyzed for each node. A discrete Gamma distribution was used to model evolutionary rate differences among the sites.

### 2.2. Ribosomal Genes

Non-coding loci are frequently utilized in phylogenetic investigations for species differentiation, including the internal transcribed spacer regions (ITS-1 and ITS-2) and the 18S and 28S ribosomal genes [24,38,45,46]. In a previous study, Zahler et al. [47] utilized a 274base pair segment of the ITS-2 sequence to establish the conspecific nature of six closely related tick species within the *R. sanguineus* complex. These species share noteworthy similarities in phenotype, morphology, and genetic traits. Barker [48] examined the ITS-2 region in 16 populations of *Rhipicephaline* ticks from various countries, including *R. microplus* from Australia, Kenya, South Africa, and Brazil; *R. decoloratus* and *R. evertsi* from Kenya; *R. appendiculatus* from Kenya, Zimbabwe, and Zambia; and *R. zambesiensis* from Zimbabwe. The results revealed unique ITS-2 genes in all 16 populations, with nucleotide variations observed at both the species and genus levels. This differentiation allowed distinguishing populations and species within the *Boophilus* and *Rhipicephalus* genera.

The ITS-2 gene demonstrated efficacy in discriminating between *R. appendiculatus* and *R. zambesiensis*, two closely related species with morphological similarities. In a study by Guzman et al. [49], intraspecific genetic distances were characterized between two Cuban strains of *R. microplus*, compared with Mexican reference ticks, using the ITS-2 gene. The findings indicated that both tick populations were *R. microplus* species, with intraspecific and interspecific differences ranging from 0% to 1% and 0% to 2%, respectively. However, it is worth noting that the ITS-2 marker did not support the previously described phylogenetic relationships within the *R. microplus* complex clades A, B, or C, as outlined by Burger et al. [17] and Low et al. [18].

In South Africa, researchers utilized the nuclear ITS-2 marker to explore the genetic relationship between *R. microplus* and *R. decoloratus* ticks. Sequence analyses revealed that *R. decoloratus* differs from *R. microplus* in 97 nucleotide substitutions and numerous polymorphisms. However, recombination of ITS-2 could not be detected in either species, and the species complex could not be separated into its respective clades [23]. Consequently, the study concluded that while ITS-2 is helpful in assessing interspecific variation, it lacks the resolution required for intraspecific variations. The ITS-2 nucleotide sequence of *R. microplus* from Pakistan (MZ542565) shows that it is clustered together with those of Chinese, Indian, and other Pakistani strains, providing strong support for the monophyly of the *R. microplus* complex. Furthermore, *R. microplus* strains from Pakistan (MW580928 and MW580866) display no genetic variation (100% similarity) with Chinese strains (MK224585; MK224584; and KX450289) or Indian (MK621182; MH598985; and MF946462) strains. The phylogenetic tree also revealed clustering of most of the other *Rhipicephalus* species, with a clade consisting of *R. bursa* and sister clades of *R. zambeziensis, R. appendiculatus*, *R. turanicus*, *R. sanguineus*, and *R. guilhoni* [50]. In another study, the *R. microplus* species complex from Bangladesh, Myanmar, and Pakistan was subjected to phylogenetic analysis using the ITS-2 gene. The results confirmed that the *R. microplus* complex comprises at least five distinct taxa, with ticks from Bangladesh, Myanmar, and Pakistan belonging to *R. microplus* clade C [27]. TheITS-2-based sequences from Pakistan, China, and India were grouped into a single clade of *R. microplus*, indicating consistency in interspecies variations. To assess the genetic diversity between *R. microplus* strains at the global level, various *R. microplus* GenBank sequences were retrieved, aligned, and used to construct a phylogenetic tree based on the ITS-2 gene. The *Hyalomma anatolicum* ITS-2 region was employed as an outgroup (Supplementary Figures S1 and S2). The analysis revealed genetic diversity ranging from 0.1% to 2.9% among the Indian *R. microplus* strains (Supplementary Figure S3). Consequently, the ITS-2 gene appears to be suitable for assessing interspecificity but needs the resolution to distinguish intraspecific variations.

Few researchers have used the 18S rDNA gene for genetic diversity studies. Black et al. [45] studied the 18S rDNA gene in all tick subfamilies and found that Hyalomminae ticks are clustered with members of the Rhipicephalinae subfamily in the phylogenetic tree. In addition to this, Mangold et al. [46] reported low genetic variation among closely related species based on the 18S rDNA gene among six European hard tick genera (*R. annulatus*, *R. pusillus*, *Dermacentor marginatus*, *H. lusitanicum*, *Haemaphysalis punctata*, and *Ixodes ricinus*). In another study on Australian endemic hard ticks (Ixodidae family), including Rhipicephalinae ticks, it was observed that only Amblyomminae were paraphyletic in the group [51]. These findings suggest that the 18S rRNA gene may not be suitable for studying closely related tick genera but may perform well for grouping taxa.

Norris et al. [52] sequenced domain III of the 12S rDNA gene of 51 tick species, and the sequences were compared with previously published sequences [53] and newly sequenced 16S sequences. Both the 12S and 16S genes yielded poorly resolved trees due to the high AT content in the mitochondrial genome, resulting in significant homoplasy. These genes might be more suitable for resolving relationships at the intraspecific level or among closely related taxa. Few studies have reported that the genus *Rhipicephalus* is closely clustered with the *R. evertsi* and *R. pravus* species based on the 12S rDNA gene [54,55]. In a recent study, the 16S rRNA gene was used to characterize five tick species (*R. microplus*, *R. sanguineus*, *R. haemaphysaloides*, *H. cornigera*, and *H. mageshimaensis*) from Hainan Island, China. The results revealed that the *R. microplus* Hainan Island strains formed a clade (clade B) within the *R. microplus* complex, which is closely related to strains from Thailand, China, Taiwan, Malaysia, Africa, and South America. The genetic diversity within and between the groups was minimal, ranging from 0.012 to 0.048 and 0.000 to 0.012, respectively. The North Indian *R. microplus* strains, along with those from Pakistan, Bangladesh, Myanmar, South India (Chennai and Bengaluru), and Central India (Madhya Pradesh), formed a single clade (clade C). Moreover, the strains from Yunnan and Henan in China clustered in clade B, showing proximity to the Indian and Egyptian strains. The strains of Southeast Asia (Thailand), South America (Colombia and Mexico), and Africa (Mozambique) were arranged in a separate clade, namely clade A. All of these clades have high bootstrap values [33]. Furthermore, insignificant genetic diversity (0.01021 ± 0.00146) was observed among various *R. microplus* strains from different countries, including Pakistan, China, Indonesia, Colombia, Mexico, Thailand, Mozambique, and India [33]. Various studies have explored the utility of different genetic markers for understanding the relationships and diversity among tick species. Although the 18S rDNA gene has been found to be valuable for grouping taxa, it may not provide the necessary resolution to distinguish closely related tick genera. Similarly, the 12S and 16S rDNA genes have limitations due to the high AT content in the mitochondrial genome, making them more suitable for assessing relationships at the intraspecific level or among closely related taxa. In contrast, the ITS-2 marker has shown promise in some studies for distinguishing tick species and populations. However, it should be noted that the ITS-2 marker may not be suitable for resolving relationships at higher taxonomic levels or for closely related species. A summary of each genetic maker is presented in Supplementary Table S1. Hence, the choice of genetic marker should be tailored based on the level of taxonomic resolution and the group of ticks under investigation. Combining multiple markers and integrating additional molecular and morphological data may provide a more comprehensive understanding of tick diversity and its relationships.

### 2.3. Microsatellites

Microsatellites, or simple sequence repeats (SSRs), are brief sequences consisting of 1–9 base pairs that repeat in tandem. In contrast, minisatellites are composed of recurring core elements, typically 10–30 base pairs in length, forming tandem arrays extending up to approximately 30 KB or more. These satellite markers are widely distributed throughout the genome, known for their high polymorphism and polyallelic nature, and are extensively employed in studies focusing on population structure and genetic diversity [56,57]. Expressed sequence tags (ESTs) are a potentially valuable source of microsatellite and minisatellite markers [58].

In a pioneering effort, Chigagure et al. [59] conducted the first study on microsatellites in the economically significant tick species *R. microplus* and characterized eight polymorphic microsatellite loci for population studies. Subsequently, nine microsatellite loci were isolated and characterized within the *R. microplus* species in New Caledonia, revealing high genetic diversity ranging from 0.61 to 0.72 at seven of these loci, which makes them suitable for population genetics studies [60]. Kanduma et al. [57] also reported the presence of polymorphic micro- and minisatellite markers, which proved valuable for estimating genetic diversity within and between the tick populations, particularly amongst the *Rhipicephaline* species. Out of 66 variable number tandem repeat (VNTR) loci, 20 microsatellites and nine minisatellites exhibited genetic variation (polymorphism) within a population. These markers were characterized to distinguish populations of *R. appendiculatus* and different species of *Rhipicephalus* [57]. To assess the genetic diversity and population structure of *R. microplus*, Sungirai et al. [61] utilized eight polymorphic microsatellite loci and reported genetic diversity ranging from 0.755 to 0.802 across all the populations. Significantly, their findings revealed substantial gene flow, with 97% of the genetic variation observed within the populations. However, when utilizing 11 microsatellite repeat loci to evaluate the genetic diversity within *R. microplus* populations from diverse sources, including white-tailed deer and cattle, Busch et al. [3] did not identify significant genetic differences between these tick populations despite their sharing the same environment. This study underscores the importance of employing microsatellite markers to probe genetic diversity and relationships among tick populations.

It is important to highlight that microsatellite markers designed explicitly for *R. microplus* have exhibited variations in their flanking regions [62] in the presence of null alleles [60] and have encountered challenges related to amplification [3]. Despite these challenges, micro- and minisatellites have proven advantages in studying tick populations and genetic diversity. Due to their highly polymorphic nature, these markers are often successfully applied to various tick species. Although micro- and minisatellites provide valuable insights into tick genetics, it is crucial to acknowledge that their effectiveness can vary in the presence of null alleles and marker variations. In summary, microsatellite and minisatellite data contribute significantly to our understanding of a tick population’s structure, which plays a pivotal role in developing effective tick management strategies.

## 3. Diversity in Bm86 Gene

After the commercialization of TickGARD and Gavac in Australia and Central American countries, attempts were made to control *R. microplus* using these vaccines in integrated management format. TickGARD and Gavac contain the recombinant *R. microplus* Bm86 gut antigen expressed in *Escherichia coli* and *Pichia pastoris*, respectively [37,38]. Immunization of animals reduced the number of engorged female ticks, their weight, and their reproductive capacity. Thus, following immunization, a reduction in pasture contamination by the larvae resulted in a reduction in the severity of infestations in subsequent generations being observed. However, field trials in various countries have shown varying levels of efficacy. In addition, they were found ineffective against some of the strains of *R. microplus* [38]. The genetic polymorphism in the Bm86 gene amongst *R. microplus* populations globally was implicated as one of the reasons for the reduced efficacy of commercial anti-*R. microplus* vaccines [37,38,39,40,41,42]. In India, phylogenetic analysis of the nucleotide and amino acid sequences of the Indian Veterinary Research Institute strain IVRI-I Bm86 and published global reference Bm86 sequences revealed that IVRI-I Bm86 is evolutionarily closely related to the Thailand and Pakistan strains and is distant from commercial vaccine strains (Yeerongpilly, Mexico, and others) [39]. This may be due to the geographical locations of the Thailand and Pakistan strains, which are geographically closer than those of other countries, and these data are in agreement with the previous observations by Kaewmongkol et al. [40]. Sequence identity matrix analysis showed that the IVRI-I Bm86 protein has 93% sequence identity (7% divergence) with the Yeerongpilly (TickGARD^TM^) vaccine strain [39]. A divergence level of more than 2.8% has been reported as a limiting factor in the variation of efficacy of Bm86-based vaccines [40,41]. The sequence divergence data validate the earlier observations in which 44.5% and 25.1% efficacy against *R. microplus* (IVRI-I strain) and *H. anatolicum* (IVRI-II strain), respectively, were recorded in a pen trial using a commercial Cuban Bm86 vaccine [42]. The high diversity of IVRI-I Bm86 and low efficacy of the commercial Mexican Bm86 vaccine in India provided significant insight into the development of region strain-specific vaccines. Even within the region, variation in the nucleotide and amino acid sequence of Bm86 in *R. microplus* strains showed 95.6–99.8% and 93.2–99.5% identity in nucleotide and amino acid sequences, respectively [39,40,41]. Specific amino acid substitutions or mutations in the conserved sequences of Bm86 indifferent Indian strains were recorded (Table 1). A significant level of polymorphism among Bm86 may have resulted from adapting the tick species to different climatic conditions and cattle breeds. The *R. microplus* strains from various regions have experienced different environmental pressures, which may have influenced the physiological, morphological, and genetic variations among these strains. The results indicate that there is a need to identify conserved vaccine candidates in economically important tick species for the development of effective anti-tick vaccines.

## 4. Diversity in Sodium Channel Genes

The invasive potentiality of *R. microplus* is not only attributed to adaptability to any agroclimatic conditions [5,7] but also to a capacity for speedy development of resistance to major classes of acaricides [63,64,65,66]. Acaricide resistance is a pre-adaptive phenomenon where the rare allele is pronounced upon treatment pressure. Thus, the resistant allele of *R. microplus* evades the acaricide’s effects successfully. Mutations in the acaricide targets are a primary mechanism of developing adaptive resistance in ticks. Non-synonymous mutation(s) leads to structural and conformational changes in the acaricide target(s), rendering the target site(s) non-responsive to the acaricides [67]. Genetic changes within a tick population can also result in raised metabolism, such as for cytochrome P450s, esterases, and glutathione S-transferases, or sequestration of the acaricide [68]. Understanding the changes in acaricide resistance genes that express the inherited resistance character (trait) is essential for early diagnosis and formulating control strategies. It also provides valuable information on the population structure, gene flow, and evolutionary adaptation [69]. Synthetic pyrethroids, amidines, and macrocyclic lactones are the major groups of acaricides used for tick management compared with other acaricides. Consequently, studies on possible molecular mechanisms of the development of resistance to this group of acaricides are mostly attempted with a high level of variation. Although the same acaricide was used in different countries to control *R. microplus*, point mutations at different locations in the targeted gene of *R. microplus* strains were reported. We reviewed only the diversity in the gene involved in conferring resistance against synthetic pyrethroids, as the reported mutations in other acaricides’ targeted genes could not phenotypically show any association with resistance.

### Sodium Channel: Synthetic Pyrethroid Resistance

Synthetic pyrethroids are widely used to control ticks in general. Pyrethrins and pyrethroids are potent neurotoxins that eliminate ticks by acting on sodium ion channels. They induce variations in nerve membrane permeability to sodium (Na+) and potassium (K+) ions, leading to nerve excitation [70]. Deltamethrin, cypermethrin, flumethrin, and fenvalerate are the synthetic pyrethroids (SPs) widely used against tick populations.

Due to its indiscriminate and unregulated use, various tick researchers have observed resistance to this compound [71,72,73,74]. Research efforts to reveal the correlation between resistance to SPs and the changes in sodium channel genes at the molecular level were undertaken, and mutations (mainly SNPs) in the resistant population of *R. microplus* were reported [75,76,77,78,79]. Among the two types of mutations—synonymous and non-synonymous—the non-synonymous one contributes to a significant relationship with the resistance to SP compounds. As mentioned in Table 2, five non-synonymous SNPs (T2134A, T2134C, G215T, C190A, and T170C) exhibited a reliable association with resistance to pyrethroids [76,77,78,80,81]. In India, mutation at nucleotide position 190, where CTC changes to ATC in the domain II S4-5 linker region, was reported [82,83,84,85]. Identification of the specific changes in proteins and their effects was a breakthrough observation in the resistance characterization study of *R. microplus* against SP compounds. Many researchers subsequently characterized pyrethroid resistance in *R. microplus* [85,86,87]. While working on synthetic pyrethroid resistance, Nagar et al. [82] could not describe either G215 T or putative superkdr T170C substitutions in field strains or the reference-resistant strain of *R. microplus*. Further studies on the sodium channel are needed to confirm the presence or absence of different mutations and provide clear evidence regarding potential molecular markers for genotyping pyrethroid resistance.

## 5. Conclusions

Conclusively, it was observed that the literature on genetic diversity among rDNA, mitochondrial genes, satellites genes, Bm86, and in the targeted gene of SP insecticide of *R. microplus* reported high levels of polymorphisms in the *R. microplus* species complex. It is evident that these variabilities correlate well with the capability of adaptation, effective transmission of pathogens, and higher reproductive capacity of *R. microplus*. Polymorphisms were enormously higher not only in inter-strain but also intra-strain situations. Analysis of the data of various genes bestowed apprehension in the population structure, lineages, and haplotyping of *R. microplus* and provided a baseline for subsequent work on the establishment of linkage between genetic variability and phenotypic characters. Broad genetic analyses are required to be carried out throughout endemic areas by joining various groups working on genetic variability together in a consortium mode to gain a deep understanding of the implication of variability in biology, evolution, and population structure and develop an integrated strategy for the management of cattle ticks.

## Figures and Tables

**Table 1:** Specific amino acid substitutions or mutations in the conserved sequence of the IVRI-I Bm86 gene in different *R. microplus* Indian strains.

State	Strains	Amino Acid Changes with Respect to IVRI-I Bm86 Conserved Sequence
Assam	Nagaon	N442D, I590V, K595N
	Barpeta	N442D, A499T, D500N, G562D, H563R, I590V, K595N
	Kamrup	N442D, D500N, G562D, E568Q, K595N, A614P
	Sonitpur	N442D, A499T, D500N, G562Y, H563R, R567G, I590V, K595N
	Morigaon	N442D, A499T, D500N, G562D, I590V
	Dibrugarh	N442D, A499T, D500N, G562D, I590V, K595N
Rajasthan	Alwar	N442D, A499T, D500N, E508K, G562D, H563R, R567G, I590V, K595N
	Sikar	N442D, A499T, D500N, G562D, H563R, R567G, I590V, K595N
	Jaipur	N442D, A499T, D500N, G562D, H563R, R567G, I590V, K595N
	Chittorgarh	N442D, A499T, D500N, K521R, G562D, H563R, S566F, I590V, K595N
	Pratapgarh	N442D, D500N, G562D, K595N, A614P
	Bharatpur	N442D, D500N, G562D, K595N, A614P
	Banswara	N442D, D500N, G562D, K595N, A614P
	Bhilwara	N442D, A499T, D500N, D519G, G562D, H563R, I590V, K595N
	Churu	N442D, A499T, D500N, G562D, H563R, R567G, I590V, N593D, K595N
	Dausa	N442D, A499T, D500S, K521R, K554I, G562D, R567G, I590V, E603D, A614S
	Udaipur	N442D, N459F, A499T, D500N, K86R, G562D, H563R, S566F, I590V, K595N
	Dungarpur	N442D, L459F, A499T, D500N, D84G, K86R, G562D, S566F, K595N
Maharashtra	Jalgaon	N442D, L459F, A499T, D500N, E89G, G562D, H563R, S566F, I590V, K595N
	Nashik	N442D, D500N, G562D, E133Q, K595N, A614P
	Dhule	N442D, A499T, D500N, G562D, I590V, K595N, A614P
	Ahmednagar	N442D, A499T, D500N, E524G, G562D, E568Q, K595N, A614P
	Raigad	N442D, C464R, D500N, G562D, H563R, R567G, I590V, K595N
	Pune	N442D, A499T, D500N, G562D, H563R, S566F, I590V, K595N, K601T, D618N
	Aurangabad	N442D, D500N, E508K, G562Y, H563R, R567G, I590V, K595N
	Satara	N442D, A499T, D500N, G562Y, H563R, R567G, I590V, K601T, D616N
	Solapur	N442D, A499T, D500N, G562Y, H563R, R567G, I590V, K601T, D616N
Madhya Pradesh	Khandwa	N442D
	Shajapur	N442D, A499T, D500N, G562D, H563R, R567G, I590V, K595N
	Barwani	N442D, A499T, D500N, G562D, H563R, S566F, R567G, I590V, K595N
	Mandsaur	N442D, A499T, D500N, G562D, H563R, R567G, I590V, K595N
	Indore	N442D, A499T, D500N, G562D, H563R, R567G, I590V, K595N
	Ujjain	N442D, A499T, D500N, G562D, H563R, R567G, I590V, K595N
Uttar Pradesh	Pilibhit	N442D, D500N, D618N
	Raebareli	N7D, D500N, G562D, H563R, S566F, I590V, K595N, E166T
	Lucknow	N442D, D500N, G562D, H563R, S566F, I590V, K595N, E166T
Haryana	Panipat	N442D, A499T, D500N, G562D, H563R, R567G, S575G, I590V, K595N
	Sonipat	N442D, F460I, S482F, A499D, D500N, G562D, R567G, S575G, I590V, K595N
	Kurukshetra	N442D, D500N, G562D, H563R, R567G, S575G, I590V, K595N
	Yamuna Nagar	N442D
	Kaithal	N442D, F460Y, G467D
	Ambala	N442D, A499T, D500N, E508K, G562D, H563R, R567E, D578G, I590V, K595N, V597A
	Karnal	N442D, A499T, D500N, G562D, H563R, R567G, I590V, K595N
	Hisar	N442D, A499T, D500N, G562D, H563R, S566F, I590V, K595N
	Fatehabad	N442D, T469K, E473G, A608S
	Bhiwani	N442D, A499T, D500N, G562D, H563R, I590V, K595N
	Rohtak	N442D, A499T, D500N
Uttarakhand	Mukteswar	N442D, A499T, D500N, G562Y, H563R, R567G, E577V, I590V, K595N
	Haridwar	N442D, A499T, D500N, G562Y, H563R, R567G, E577V, I590V, K595N
	New Tehri	N442D, A499T, D500N, G562Y, H563R, R567G, E577V, I590V, K595N, K601T
	Uttarkashi	N442D, A499T, D500N, E508K, G562Y, H563R, R567G, E577V, I590V, K595N
	Dehradun	N442D, A499T, D500N, G562Y, H563R, R567G, E568Q, I590V, K595N
	Almora	N442D, A499T, D500N, G562Y, H563R, R567G, E577V, I590V, K595N, K601T
Gujarat	Ahmedabad	N442D, V483M, A499T, D500N, G562D, H563R, I590V, K595N, D616N
	Junagadh	N442D, V483M, A499T, D500N, G562D, H563R, R567G, I590V, K595N, D616N
	Porbandar	N442D, V483M, A499T, D500N, G562D, H563R, I590V, K595N, D616N
	Jamnagar	N442D, V483M, A499T, D500N, K86R, G12D, H563R, I590V, K595N, D616N
	Somnath	N442D, V483M, A499T, D500N, G562D, H563R, I590V, K595N, D616N
	Bhavnagar	N442D, V483M, A499T, D500N, E508K, G562D, H563R, I590V, K595N, D616N
	Anand	N442D, V483M, A499T, D500N, G562D, H563R, I590V, K595N, D616N
Punjab	Muktsar	N442D, A499T, D500N, I590V
	Firozpur	N442D, A499T, D500N, K595N
	Ludhiana	N442D, A499T, D500N, H128D
	Mansa	N442D, A499T, D500N, I590V
	Moga	N442D, A499T, D500N, G562D

**Table 2:** Single-nucleotide polymorphisms (SNPs) in the sodium channel gene of *R. microplus*.

Location	Position of Nucleotide Substitution	Position of Amino Acid Substitution	Country	References
Domain III S6	T2134A	F1550I	Mexico and USA	[75,76,80,88,89,90,91,92]
Domain II S4-5 linker	C190A	L64I	Australia	[77,78,90,92]
			Brazil	[92,93,94,95,96]
			Mexico	[80]
			USA	[92,97]
			Africa	[98]
			Argentina	[92]
			Columbia	[99]
			India	[73,81,82,86,87]
Domain II S4-5 linker	G215T	G72V	Australia	[78,92]
			Sri Lanka	[100]
			Mexico	[79]
Domain II S4-5 linker	T170C	M57T	Mexico	[79,80] *
			USA	[80]
			Colombia	[99]
Domain III S6	T2134C	F1550L	Colombia	[99,101] *

* Researchers who described SNPs for the first time.

## Data Availability

Data sets used or analyzed during the present study are available from the corresponding author upon reasonable request.

## Supplementary Materials

The following supporting information can be downloaded at https://www.mdpi.com/article/10.3390/pathogens13060516/s1. Figure S1: Phylogenetic tree of global *Rhipicephalus microplus* strains based on ITS-2 gene. Figure S2: Sequence identity matrix of global *R. microplus* strains based on ITS-2 gene. Figure S3: Sequence identity matrix of Indian *R. microplus* strains based on ITS-2 gene. Table S1: Summary of roles and limitations of genetic markers in tick diversity studies.
