# Supplementary material for: Genetic Diversity of Rhipicephalus (Boophilus) microplus for a Global Scenario: A Comprehensive Review
Source: Pathogens. 2024 Jun 18;13(6):516. doi: 10.3390/pathogens13060516 (PMC11206262; doi:10.3390/pathogens13060516)
Supplement: Supplementary file 1 [file pathogens-13-00516-s001.zip › pathogens-3017578-supplementary/pathogens-3017578-figures.pdf]

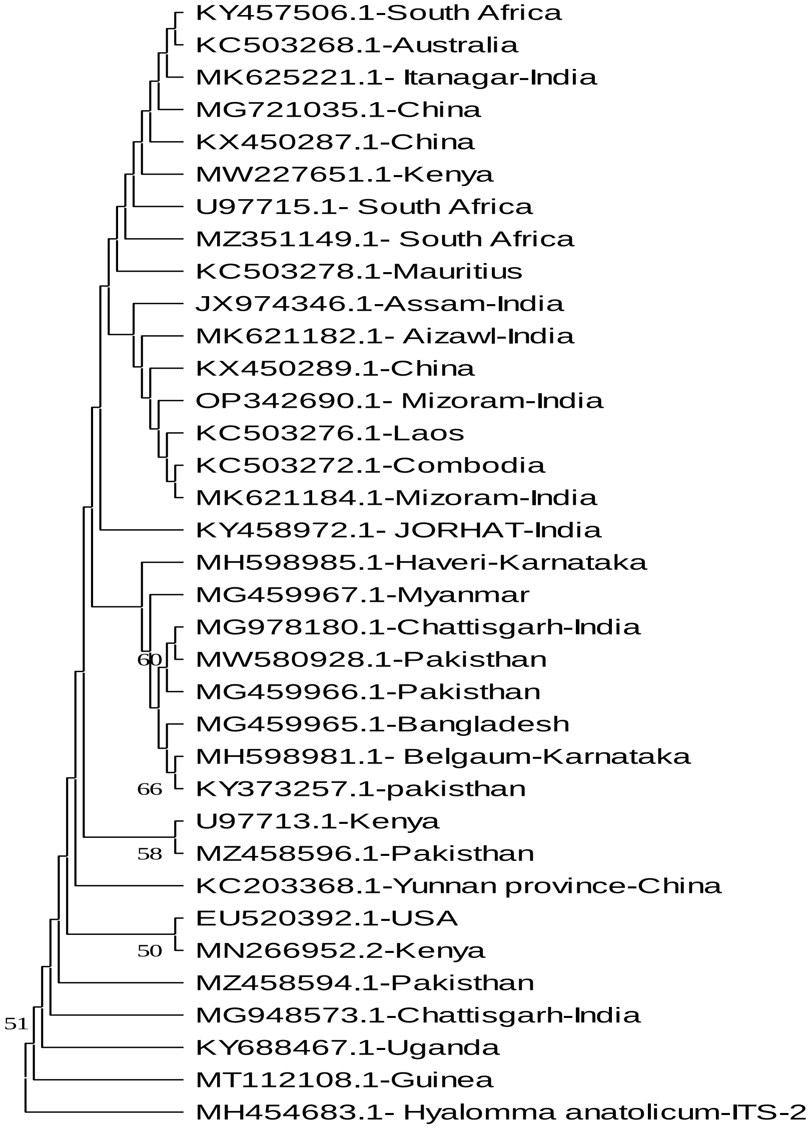

Supplementary Figure S1: Phylogenetic tree of global *Rhipicephalus microplus* isolates based on ITS-2 gene

[The evolutionary history was inferred using the Maximum-likelihood method. The optimal tree is shown. The percentage of replicate trees in which the associated taxa clustered together in the bootstrap test (1000 replicates) are shown below the branches. The evolutionary distances were computed using the Tamura-Nei method and are in the units of the number of base substitutions per site. The rate variation among sites was modeled with a gamma distribution (shape parameter = 1). This analysis involved 35 nucleotide sequences. Codon positions included were 1st+2nd+3rd+Noncoding. All ambiguous positions were removed for each sequence pair (pairwise deletion option). There were a total of 1207 positions in the final dataset. Evolutionary analyses were conducted in MEGA11]

Percent Identity

Divergence

	1	2	3	4	5	6	7	8	9	10	11	12	13	14	15	16	17	18	19	20	21	22	23	24	25	26		
**1**		98.3	85.9	96.8	98.8	98.7	60.0	59.9	98.7	90.8	90.8	59.8	59.9	82.0	80.8	59.6	62.7	98.7	98.7	98.8	76.2	85.9	98.3	97.6	98.7	9.9	**1**	EU520392.1-USA-1.seq
**2**	1.7		84.9	95.8	99.4	99.3	59.3	59.2	99.3	89.9	89.9	59.2	59.2	81.2	79.9	59.0	62.0	99.3	99.2	99.4	75.3	84.9	98.3	96.5	99.3	10.0	**2**	KC503268.1-Australia.seq
**3**	15.5	16.7		84.2	85.3	85.4	59.8	59.7	85.4	82.7	82.7	59.6	59.7	79.0	78.3	59.5	62.5	85.4	85.2	85.3	75.9	100.0	84.8	85.0	85.4	9.9	**3**	KC503272.1-Combodia.seq
**4**	0.8	1.2	14.5		99.0	98.9	60.7	60.7	98.9	91.6	91.6	60.6	60.7	82.9	81.6	60.3	63.4	98.9	98.8	99.0	76.9	86.5	98.5	98.7	98.9	10.1	**4**	KC503278.1-Mauritius.seq
**5**	1.2	0.5	16.3	0.7		99.9	59.7	59.6	99.9	90.4	90.4	59.5	59.6	81.7	80.4	59.4	62.4	99.9	99.8	100.0	75.8	85.3	98.9	97.1	99.9	9.8	**5**	KX450287.1-China.seq
**6**	1.3	0.6	16.1	0.8	0.1		59.8	59.7	100.0	90.3	90.3	59.6	59.7	81.7	80.5	59.5	62.5	100.0	99.7	99.9	75.9	85.4	98.8	97.0	100.0	9.9	**6**	KX450289.1-China.seq
**7**	0.3	0.7	0.0	0.8	0.1	0.0		99.7	100.0	95.8	95.8	99.7	99.7	95.1	94.8	94.8	97.1	100.0	99.6	99.9	100.0	100.0	99.3	99.9	100.0	16.6	**7**	KY373257.1-pakisthan.seq
**8**	0.3	0.7	0.0	0.7	0.1	0.0	0.0		100.0	95.7	95.7	99.9	100.0	95.0	94.7	94.7	96.9	100.0	99.6	99.9	100.0	100.0	99.3	99.9	100.0	16.6	**8**	MG459966.1-Pakisthan.seq
**9**	1.3	0.6	16.1	0.8	0.1	0.0	0.0	0.0		90.3	90.3	59.6	59.7	81.7	80.5	59.5	62.5	100.0	99.7	99.9	75.9	85.4	98.8	97.0	100.0	9.9	**9**	JX974346.1-Assam-India.seq
**10**	7.4	7.7	16.5	7.4	7.2	7.3	3.3	3.3	7.3		100.0	58.7	58.7	83.5	82.3	60.8	63.0	92.8	92.7	92.9	75.2	84.9	92.5	92.5	92.8	10.1	**10**	KY688467.1-Uganda.seq
**11**	7.4	7.7	16.5	7.4	7.2	7.3	3.3	3.3	7.3	0.0		58.7	58.7	83.5	82.3	60.8	63.0	92.8	92.7	92.9	75.2	84.9	92.5	92.5	92.8	10.1	**11**	KY688467.1-Uganda-1.seq
**12**	0.3	0.7	0.0	0.7	0.1	0.0	0.0	0.0	0.0	3.3	3.3		100.0	95.1	94.8	94.8	97.1	100.0	99.6	99.9	100.0	100.0	99.3	99.9	100.0	16.6	**12**	MG459965.1-Bangladesh.seq
**13**	0.3	0.7	0.0	0.7	0.1	0.0	0.0	0.0	0.0	3.3	3.3	0.0		95.0	94.7	94.7	96.9	100.0	99.6	99.9	100.0	100.0	99.3	99.9	100.0	16.6	**13**	MG459967.1-Myanmar.seq
**14**	0.2	0.6	3.3	0.9	0.1	0.0	0.0	0.0	0.0	0.5	0.5	0.0	0.0		98.5	72.8	75.0	100.0	99.7	99.9	89.2	96.6	99.4	99.9	100.0	12.1	**14**	MG948573.1-Chattisgarh-India.seq
**15**	1.1	1.6	3.6	1.9	1.0	0.9	0.0	0.0	0.9	1.2	1.2	0.0	0.0	0.9		73.1	75.2	99.0	98.7	98.9	89.3	96.3	98.4	98.9	99.0	12.2	**15**	MG978180.1-Chattisgarh-India.seq
**16**	2.8	3.2	2.5	3.4	2.6	2.5	0.0	0.0	2.5	2.9	2.9	0.0	0.0	2.5	2.5		97.5	97.5	97.1	97.4	97.5	97.5	96.9	97.4	97.5	16.3	**16**	MH598981.1- Belgaum-Karnataka.seq
**17**	0.3	0.7	0.0	0.8	0.1	0.0	0.0	0.0	0.0	1.9	1.9	0.0	0.0	0.0	0.0	2.5		100.0	99.6	99.9	100.0	100.0	99.3	99.9	100.0	15.9	**17**	MH598985.1-Haveri-Karnataka.seq
**18**	1.3	0.6	16.1	0.8	0.1	0.0	0.0	0.0	0.0	7.3	7.3	0.0	0.0	0.0	0.9	2.5	0.0		99.7	99.9	75.9	85.4	98.8	97.0	100.0	9.9	**18**	MK621182.1- Aizawl-India.seq
**19**	1.4	0.7	16.5	0.9	0.2	0.3	0.4	0.4	0.3	7.4	7.4	0.4	0.4	0.3	1.2	2.9	0.4	0.3		99.8	75.6	85.2	98.7	96.9	99.7	9.8	**19**	MK625221.1- Itanagar-India.seq
**20**	1.2	0.5	16.3	0.7	0.0	0.1	0.1	0.1	0.1	7.2	7.2	0.1	0.1	0.1	1.0	2.6	0.1	0.1	0.2		75.8	85.3	98.9	97.1	99.9	9.8	**20**	MW227651.1-Kenya.seq
**21**	0.2	0.7	0.0	1.0	0.1	0.0	0.0	0.0	0.0	2.6	2.6	0.0	0.0	0.0	0.1	2.5	0.0	0.0	0.3	0.1		100.0	99.5	99.9	100.0	13.1	**21**	MT112108.1-Guinea.seq
**22**	15.6	16.8	0.0	14.6	16.3	16.2	0.0	0.0	16.2	16.6	16.6	0.0	0.0	3.4	3.7	2.5	0.0	16.2	16.6	16.3	0.0		84.8	84.9	85.3	9.9	**22**	KY458972.1- JORHAT-India.seq
**23**	1.2	0.5	15.7	0.7	0.0	0.1	0.1	0.1	0.1	7.2	7.2	0.1	0.1	0.1	1.0	2.7	0.1	0.1	0.2	0.0	0.1	15.8		97.7	99.9	9.9	**23**	U97715.1- South Africa.seq
**24**	0.1	0.5	13.5	0.7	0.0	0.1	0.1	0.1	0.1	6.6	6.6	0.1	0.1	0.1	1.0	2.6	0.1	0.1	0.2	0.0	0.1	13.6	0.0		99.8	10.1	**24**	MZ351149.1- South Africa.seq
**25**	1.3	0.6	16.1	0.8	0.1	0.0	0.0	0.0	0.0	7.3	7.3	0.0	0.0	0.0	0.9	2.5	0.0	0.0	0.3	0.1	0.0	16.2	0.1	0.1		9.9	**25**	KC503276.1-Laos.seq
**26**	88.0	85.1	86.7	87.5	88.0	86.7	86.7	86.7	86.7	88.0	88.0	86.7	86.7	86.7	86.7	86.7	86.7	86.7	88.0	88.0	86.7	86.7	88.0	88.0	86.7		**26**	MH454683.1- Hyalomma anatolicum-IT
	**1**	**2**	**3**	**4**	**5**	**6**	**7**	**8**	**9**	**10**	**11**	**12**	**13**	**14**	**15**	**16**	**17**	**18**	**19**	**20**	**21**	**22**	**23**	**24**	**25**	**26**		

Supplementary Figure S2: Sequence identity matrix of global *R. microplus* isolates based on ITS-2 gene

Percent Identity

Divergence

	1	2	3	4	5	6	7	8	9		
1		99.7	99.7	81.5	80.2	59.2	62.2	99.7	10.0	1	MK625221.1- Itanagar-India.seq
2	0.3		99.9	81.7	80.5	59.5	62.5	100.0	10.0	2	JX974346.1-Assam-India.seq
3	0.3	0.1		81.6	80.3	59.4	62.4	99.8	10.0	3	KY458972.1- Jorhat-India.seq
4	0.3	0.0	0.1		98.5	72.8	75.0	100.0	12.2	4	MG948573.1-Chattisgarh-India.seq
5	1.2	0.9	1.1	0.9		73.1	75.2	99.0	12.3	5	MG978180.1-Chattisgarh-India.seq
6	2.9	2.5	2.5	2.5	2.5		97.5	97.5	16.7	6	MH598981.1- Belgaum-India.seq
7	0.4	0.0	0.0	0.0	0.0	2.5		100.0	16.0	7	MH598985.1-Haveri-India.seq
8	0.3	0.0	0.1	0.0	0.9	2.5	0.0		10.0	8	MK621182.1- Aizawl-India.seq
9	83.4	83.4	83.4	83.4	83.4	80.5	83.4	83.4		9	MH454683.1- Hyalomma anatolicum-ITS-2.s
	1	2	3	4	5	6	7	8	9		

Supplementary Figure S3. Sequence identity matrix of Indian *R. microplus* isolates based on ITS-2 gene
