# Supplementary material for: Genetic Diversity of Rhipicephalus (Boophilus) microplus for a Global Scenario: A Comprehensive Review
Source: Pathogens. 2024 Jun 18;13(6):516. doi: 10.3390/pathogens13060516 (PMC11206262; doi:10.3390/pathogens13060516)
Supplement: Supplementary file 1 [file pathogens-13-00516-s001.zip › pathogens-3017578-supplementary/pathogens-3017578-table.pdf]

Supplementary table1 : Summary of role and limitation of genetic markers in tick diversity studies

**Molecular Marker**	**Applications in tick diversity**	**Limitations**	**Clade Separation Effectiveness**
**ITS-2**	Useful for identifying interspecific genetic variations and differentiating between closely related species and populations.	Limited resolution for intraspecific variations<br>Does not support detailed clade separation within *R. microplus* complex	Low: Cannot effectively separate *R. microplus* clades
**18S rDNA**	Helps in grouping higher taxonomic levels, providing broad phylogenetic insights.	Low resolution for closely related species.<br>Poor for fine-scale genetic differentiation	Low: Ineffective for closely related genera
**12S rDNA**	Useful for studying relationships at intraspecific levels and among closely related taxa, despite limitations.	High AT content leading to homoplasy.<br>Poorly resolved phylogenetic trees	- Low: Limited effectiveness due to poor resolution
**16S rDNA**	Assists in characterizing tick species and understanding relationships among closely related taxa.	High AT content causing homoplasy.<br>Poor resolution in phylogenetic trees	- Low: Limited effectiveness due to poor resolution
**COX1**	widely used in DNA barcoding for species identification and genetic diversity studies.<br>species identification<br>Phylogenetic clustering	Limited resolution for intraspecific variation<br>May not detect recent population divergence	High: Successfully separates clades A, B, C, *R. australis*, and *R. annulatus*
**Microsatellites**	Highly polymorphic markers useful for studying population structure and genetic diversity, though with technical challenges.	Variations in flanking regions<br>Presence of null alleles<br>Amplification challenges	- Moderate: Provides genetic diversity insights but not detailed clade separation
